# Predictors of antibiotic use in pregnant women attending Najran University Hospital, Saudi Arabia

**DOI:** 10.15537/smj.2023.44.3.20220762

**Published:** 2023-03

**Authors:** Ali M. Alshabi, Majed S. Alshahrani, Basel A. Abdel-Wahab, Masood M. Khateeb, Ibrahim A. Shaikh, Basheerahmed A. Mannasaheb

**Affiliations:** *From the Department of Clinical Pharmacy (Alshabi); from the Department of Pharmacology (Abdel-Wahab, Khateeb, Shaikh), College of Pharmacy; from the Department of Obstetrics and Gynecology (Alshahrani), Faculty of Medicine, Najran University, Najran, and from the Department of Pharmacy Practice (Mannasaheb), College of Pharmacy, AlMaarefa University, Riyadh, Kingdom of Saudi Arabia.*

**Keywords:** antibiotics, pregnancy, complications, comorbidity, Saudi Arabia

## Abstract

**Objectives:**

To determine the prevalence of antibiotic use by pregnant women in Najran, Saudi Arabia.

**Methods:**

A total of 125 women aged 18 to 45 with a full-term pregnancy participated from October to December 2019. Age, order of current pregnancy, body mass index (BMI), history of miscarriage, and comorbidity were used to estimate antibiotic use.

**Results:**

The majority were Saudis (67.2%), aged 30-35 (39.2%) years, with no history of miscarriage (53.6%), second order of pregnancy (26.4%), and going through weeks 20-25 of pregnancy (21.6%). A total of 26.4% of pregnant women had antibiotic prescriptions in the study population. Pregnant women under 30 years were less likely to receive antibiotics.

**Conclusion:**

The results found an association between maternal age, order of pregnancy and antibiotic use during pregnancy. An association was observed between maternal BMI and the occurrence of adverse drug reactions after antibiotic use. In addition, a history of miscarriage was negatively associated with the use of antibiotics during pregnancy. These predictors of antibiotic administration have the potential to serve as general health indicators and to direct preventative strategies aimed at increasing the rational use of antibiotics.

**T**he use of medicines during pregnancy cannot be avoided entirely because some pregnant women may have acute or chronic diseases. Moreover, after the thalidomide incident in the 1960s, the use of medications, especially antibiotics during pregnancy, has come under significant scrutiny. It has been found that antibiotics are the second most commonly used medicines during pregnancy, with B-lactams and macrolide being the most commonly used.^1^ In Saudi Arabia, the usage is approximately 40% in pregnant women.^2^ There is a large amount of information on teratogenicity with regard to the type of drugs used during pregnancy, but this topic is still confusing and creates tension for pregnant women as well as their healthcare providers. Studies showcasing accountability indicate that mothers are often dismissive of birth abnormalities.^3^ The safe use of drugs during pregnancy should also be thoroughly confirmed before they are prescribed to pregnant women, and research work should be encouraged on this topic. Keeping this in mind, we caried out a study in Najran, Saudi Arabia, among pregnant women to assess their drug use during pregnancy. Women seeking prenatal care at Najran University Hospital (NUH) were surveyed on their antibiotic use during pregnancy.

## Methods

This cross-sectional included pregnant females attending the obstetrics and gynecology (Ob/Gyn) Department at NUH. Participants who were currently non-pregnant were excluded from the study. The study was carried out from October to December 2019. A total of 125 participants were chosen using convenient sampling technique.

The study tool was developed from the review of relevant literature.^4,6^ We searched well-known databases such as Scopus, PubMed, and Web of Science for similar literature. The questionnaire draft was assessed for its content validity by experts. Recommended changes were made, and a language expert translated the final draft into Arabic. It was then back-translated to English by an independent professional. A pilot study was conducted on 10 pregnant females attending the same hospital to assess the face validity. All the questions were found to be clear and understandable. Additionally, the Cronbach’s alpha was calculated (0.75), confirming the study tool’s reliability.

All pregnant women visiting NUH received the printed Arabic questionnaire. Informed consent was obtained for each individual, and participation was voluntary. The study tool was segregated into 3 sections. The first section was demographic information, including the participant’s age, nationality, body mass index (BMI), history of miscarriage, order and weeks of current pregnancy, and presence of any disease. The second part of the questionnaire was related to the prevalence of antibiotic use, such as antibiotic use during the current pregnancy, duration of use, the occurrence of adverse drug reactions, and abnormality in lab results after the period of antibiotic use. The last section was related to the type of infection suffered during the current pregnancy, such as unrinary tract infection (UTI), upper respiratory tract infections, and genital infection.

The ethical committee of Najran University approved the study (ethical approval number: 442-41-52467-DS). The investigation followed the guidelines laid out in the Helsinki Declaration.

### Statistical analysis

Data entery and analysis was carried out using the SPSS Statistics for Windows, version 21 (IBM Corp., Armonk, N.Y., USA). The significance of differences was assessed using Fisher’s exact and the likelihood ratio tests. In order to predict what factors would affect a woman’s likelihood of using antibiotics while pregnant, a regression analysis was conducted. Significant *p*-values were <0.05.

## Results

A total of 125 pregnant females visiting NUH participated in this study. The participants’ demographic characteristics are illustrated in Table 1.

**Table 1:** - Socio-demographic characteristics of the study participants (N=125).

Characteristics	n	(%)
*Age*		
<18 years	1	0.8
18-25 years	32	25.6
25-35 years	35	28.0
30-35 years	49	39.2
35-40 years	7	5.6
Above 40 years	1	0.8
*Nationality*		
Saudi	84	67.2
Indian	4	3.2
Yemeni	9	7.2
Egyptian	12	9.6
Syrian	1	0.8
Jordanian	4	3.2
Palestinian	1	0.8
Sudanese	9	7.2
Other	1	0.8
*Body mass index*		
<18	-	-
18.5 to 25	38	30.4
25-30	53	42.4

Most of the participants suffered UTI (29.6%), followed by genital infections (26.4%), and others (25.6%), during their current pregnancy. A negligible number of participants suffered gastrointestinal infections (1.6%). Although more than 50% of the participants were infected with urinary and genital infections, only one-fourth (26.4%) of them used antibiotics of some kind to self-manage these problems. Penicillin, nitrofurantoin, and cephalosporins were used by 15.2%, 6.4%, and 4.8% of the pregnant women. Almost 3-quarters (73.6%) of the participants did not use antibiotics.

Almost one-quarter (26.4%) of the pregnant females used antibiotics during their current pregnancy. Age and order of current pregnancy significantly impacted antibiotic use. Women in the 30-35 age group used significantly more antibiotics (12.8%) (*p*=0.027) compared to other age groups. Similarly, women whose current pregnancy was the fifth or more used significantly (*p*=0.039) more antibiotics compared with those whose pregnancy was less than their fourth. The independent variables had no significant association with the duration of antibiotic treatment. Pregnant women in the BMI range of 18.5-30 had a significantly (*p*=0.046) higher incidence (32%) of adverse drug reactions when compared with those with BMI >30. The detailed results are presented in Table 2.

**Table 2:** - Prevalence of antibiotic use, course of treatment, adverse drug reaction, and abnormality in lab tests after antibiotic use among pregnant women (N=125).

Characteristics	Use of antibiotics			Treatment period (course) of antibiotics				
	Yes (%)	No (%)	*P*-value	<1 week (%)	1 week (%)	2 weeks (%)	Not used (%)	*P*-value
***Age*** ^†^								
<18 years	1 (0.8)	0	0.027*	0	1 (0.8)	0	0	0.342
18-25 years	10 (8.0)	22 (17.6)		6 (4.8)	4 (3.2)	0	22 (17.6)	
25-30 years	3 (2.4)	32 (25.6)		1 (0.8)	2 (1.6)	0	32 (25.6)	
30-35 years	16 (12.8)	33 (26.4)		8 (6.4)	7 (5.6)	1 (0.8)	33 (26.4)	
35-40 years	3 (2.4)	4 (3.2)		1 (0.8)	2 (1.6)	0	4 (3.2)	
>40 years	0	1 (0.8)		0	0	0	1 (0.8)	
***Nationality*** ^†^								
Saudi	25 (20.0)	59 (47.2)	0.142	12 (9.6)	13 (10.4)	0	59 (47.2)	0.300
Indian	0	4 (3.2)		0	0	0	4 (3.2)	
Yemeni	3 (2.4)	6 (4.8)		3 (2.4)	0	0	6 (4.8)	
Egyptian	1 (0.8)	11 (8.8)		0	1 (0.8)	0	11 (8.8)	
Syrian	0	1 (0.8)		0	0	0	1 (0.8)	
Jordanian	0	4 (3.2)		0	0	0	4 (3.2)	
Palestinian	1 (0.8)	0		0	0	1 (0.8)	0	
Sudanese	3 (2.4)	6 (4.8)		1 (0.8)	2 (1.6)	0	6 (4.8)	
Others	0	1 (0.8)		0	0	0	1 (0.8)	
***Body mass index*** ^†^								
<18	-	-	0.160	-	-	-	-	0.198
18.5-25	11 (8.8)	27 (21.6)		3 (2.4)	8 (6.4)	0	27 (21.6)	
25-30	17 (13.6)	36 (28.8)		10 (8.0)	6 (4.8)	1 (0.8)	36 (28.8)	
>30	5 (4.0)	29 (23.2)		3 (2.4)	2 (1.6)	0	29 (23.2)	
***History of miscarriage*** ^§^								
Yes	16 (12.8)	42 (33.6)	0.840	7 (5.6)	9 (7.2)	0	42 (33.6)	0.586
No	17 (13.6)	50 (40.0)		9 (7.2)	7 (5.6)	1 (0.8)	50 (40.0)	
Order of current pregnancy^†^								
First	2 (1.6)	14 (11.2)	0.039*	2 (1.6)	0	0	14 (11.2)	0.159
Second	12 (9.6)	21 (16.8)		5 (4.0)	7 (5.6)	0	21 (16.8)	
Third	3 (2.4)	18 (14.4)		1 (0.8)	2 (1.6)	0	18 (14.4)	
Fourth	4 (3.2)	22 (17.6)		3 (2.4)	1 (0.8)	0	22 (17.6)	
Above fourth	12 (9.6)	17 (13.6)		5 (4.0)	6 (4.8)	1 (0.8)	17 (13.6)	
***Weeks of current pregnancy*** ^†^								
5-10 weeks	6 (4.8)	9 (7.2)	0.290	1 (0.8)	5 (4.0)	0	9 (7.2)	0.132
10-15 weeks	2 (1.6)	14 (11.2)		0	2 (1.6)	0	14 (11.2)	
15-20 weeks	9 (7.2)	15 (12.0)		5 (4.0)	3 (2.4)	1 (0.8)	15 (12.0)	
20-25 weeks	8 (6.4)	19 (15.2)		7 (5.6)	1 (0.8)	0	19 (15.2)	
25-30 weeks	4 (3.2)	17 (13.6)		2 (1.6)	2 (1.6)	0	17 (13.6)	
30-35 weeks	4 (3.2)	18 (14.4)		1 (0.8)	3 (2.4)	0	18 (14.4)	
***Presence of other disease*** ^†^								
Hypertension	0	2 (1.6)	0.535	0	0	0	2 (1.6)	0.994
Gestational diabetes	0	1 (0.8)		0	0	0	1 (0.8)	
Asthma	0	1 (0.8)		0	0	0	1 (0.8)	
Inflammatory bowel disease	0	1 (0.8)		0	0	0	1 (0.8)	
No	33 (26.4)	87 (69.6)		16 (12.8)	16 (12.8)	1 (0.8)	87 (69.6)	

Values are presented as number and percentages (%).

^†^ Likelihood ratio test,

^§^ Fisher’s exact test,

^*^ *p*<0.05

**Table 2:** - Prevalence of antibiotic use, course of treatment, adverse drug reaction, and abnormality in lab tests after antibiotic use among pregnant women (continuation) (N=125).

Characteristics	ADR occurred afteusing antibiotics?			Abnormality in lab tests afterusing antibiotics?		
	Yes (%)	No (%)	*P*-value	Yes (%)	No (%)	*P*-value
***Age*** ^†^						
<18 years	1 (0.8)	0	0.257	0	1 (0.8)	0.122
18-25 years	12 (9.6)	20 (16.0)		0	32 (25.6)	
25-30 years	12 (9.6)	23 (18.4)		5 (4.0)	30 (24.0)	
30-35 years	17 (13.6)	32 (25.6)		5 (4.0)	44 (35.2)	
35-40 years	5 (4.0)	2 (1.6)		0	7 (5.6)	
>40 years	0	1 (0.8)		0	1 (0.8)	
***Nationality*** ^†^						
Saudi	31 (24.8)	53 (42.4)	0.774	7 (5.6)	77 (61.6)	0.626
Indian	2 (1.6)	2 (1.6)		1 (0.8)	3 (2.4)	
Yemeni	4 (3.2)	5 (4.0)		0	9 (7.2)	
Egyptian	4 (3.2)	8 (6.4)		2 (1.6)	10 (8.0)	
Syrian	0	1 (0.8)		0	1 (0.8)	
Jordanian	1 (0.8)	3 (2.4)		0	4 (3.2)	
Palestinian	1 (0.8)	0		0	1 (0.8)	
Sudanese	4 (3.2)	5 (4.0)		0	9 (7.2)	
Others	0	1 (0.8)		0	1 (0.8)	
***Body mass index*** ^†^						
<18	-	-	0.046*	-	-	0.401
18.5-25	17 (13.6)	21 (16.8)		5 (4.0)	33 (26.4)	
25-30	23 (18.4)	30 (24.0)		3 (2.4)	50 (40.0)	
Above 30	7 (5.6)	27 (21.6)		2 (1.6)	32 (25.6)	
***History of miscarriage*** ^§^						
Yes	26 (20.8)	32 (25.6)	0.141	4 (3.2)	54 (43.2)	0.751
No	21 (16.8)	46 (36.8)		6 (4.8)	61 (48.8)	
***Order of current pregnancy*** ^†^						
First	5 (4.0)	11 (8.8)	0.823	1 (0.8)	15 (12.0)	0.061
Second	11 (8.8)	22 (17.6)		0	33 (26.4)	
Third	9 (7.2)	12 (9.6)		2 (1.6)	19 (15.2)	
Fourth	9 (7.2)	17 (13.6)		5 (4.0)	21 (16.8)	
Above fourth	13 (10.4)	16 (12.8)		2 (1.6)	27 (21.6)	
***Weeks of current pregnancy*** ^†^						
5-10 weeks	9 (7.2)	6 (4.8)	0.230	0	15 (12.0)	0.489
10-15 weeks	7 (5.6)	9 (7.2)		1 (0.8)	15 (12.0)	
15-20 weeks	11 (8.8)	13 (10.4)		1 (0.8)	23 (18.4)	
20-25 weeks	7 (5.6)	20 (16.0)		3 (2.4)	24 (19.2)	
25-30 weeks	7 (5.6)	14 (11.2)		3 (2.4)	18 (14.4)	
30-35 weeks	6 (4.8)	16 (12.8)		2 (1.6)	20 (16.0)	
***Presence of other disease*** ^†^						
Hypertension	2 (1.6)	0	0.099	0	2 (1.6)	0.218
Gestational diabetes	0	1 (0.8)		0	1 (0.8)	
Asthma	0	1 (0.8)		0	1 (0.8)	
Inflammatory bowel disease	1 (0.8)	0		1 (0.8)	0	
No	44 (35.2)	76 (60.8)		9 (7.2)	111 (88.8)	

Values are presented as number and percentages (%).

^†^ Likelihood ratio test,

^§^ Fisher’s exact test,

^*^ *p*<0.05, ADR: adverse drug reaction

The significant independent variables were subjected to multivariate regression analysis to determine individual predictors of antibiotic use in pregnancy. After the fine-tuning of potential confounding factors, pregnancy order was found to be a significant negative independent predictor of antibiotic use during pregnancy. First, second, and fourth pregnancies were negatively associated with antibiotic use during pregnancy in comparison to pregnancies beyond the fourth. This indicates that women pregnant <4 times are less likely to use antibiotics during pregnancy than women who have been pregnant >4 times. Similarly, a history of miscarriage was negatively associated with the use of antibiotics during pregnancy. Likewise, being below 30 years of age and at the pregnancy stage of 10-15 weeks were negatively linked with the use of antibiotics (Table 3).

**Table 3:** - Multivariate logistic regression analysis identifying the variables associated with the use of antibiotics during pregnancy.

Independent variables	Variable coefficient (B)	*P*-value	OR (95% CI) adjusted^^*^^
Did you use antibiotics during pregnancy? (YES)			
***Age***			
Below 30 years	-0.726	0.183	0.484 (0.166-0.1.41)
Above 30 years	-	-	1.00
***Nationality***			
Saudi	0.513	0.370	1.670 (0.545-5.120)
Non-Saudi	-	-	1.00
Body mass index			
18.5 to 25	1.188	0.093	3.28 (0.821-13.103)
25-30	1.223	0.069	3.398 (0.910-12.69)
Above 30	-	-	1.00
***History of miscarriage***			
Yes	-0.548	0.319	0.578 (0.196-1.700)
No	-	-	1.00
***Order of current pregnancy***			
First	-2.064	0.049	0.127 (0.016-1.011) *
Second	-0.364	0.637	0.695 (0.154-3.143)
Third	-1.995	0.031	0.136 (0.022-0.829) *
Fourth	-1.558	0.049	0.210 (0.044-0.966) *
Above 4^th^	-	-	1.00
***Weeks of current pregnancy***			
5-10 weeks	1.396	0.113	4.040 (0.718-22.728)
10-15 weeks	-0.467	0.648	0.627 (0.085-4.639)
15-20 weeks	0.973	0.220	2.647 (0.559-12.542)
20-25 weeks	1.411	0.076	4.100 (0.861-19.515)
25-30 weeks	0.389	0.658	1.475 (0.263-8.266)
30-35 weeks	-	-	1.00

* *p*<0.05, CI: confidence interval, OR: oddd ratio

## Discussion

Pregnant women who take antibiotics are more likely to give birth to a newborn with a congenital disorder. In the current study, we explored the trend towards use of antibiotics by pregnant women visiting the NUH and their concern towards use of antibiotics during their pregnancies in fear that they might cause many adverse effects on the fetus. The present study found that approximately 25% of pregnant women received antibiotics. In comparison to a research carried out in Germany, this rate of antibiotic use was significantly greater, which reported that 14.7% of pregnant women had antibiotic prescriptions.^4^ Another study carried out in Copenhagen, Denmark, revealed that the prevalence of antibiotic use was 37% during pregnancy and 33% intrapartum.^5^ However, the findings of the current study were in contrast to another study conducted in rural Ghana, which reported that a much higher percentage (65%) of pregnant women received antibiotics.^6^

The large majority of pregnant women in this study did not use antibiotics during their current pregnancy, and of those who used them, this was during the third trimester (35%); the infections that are most common among pregnant women were UTIs (29.6%) and genital infections (26.4%). Our findings were in line with the German study, which reported UTIs to be the most prevalent bacterial infection.^4^ During pregnancy, the uterus expands and prevents the bladder from releasing the flow of urine, this produces a vulnerable medium for infections that can lead to a natural prepotency of UTIs in pregnant women.^7^

Vaginal infection was discovered to be the second most prevalent bacterial infection, as hormonal imbalances occur during pregnancy and may lead to substantial lapse of immunity.^8^ Pregnant women often develop life-threatening infections, including UTIs. In pregnant women, UTIs accounted for 3.5% of all antenatal admissions, and pyelonephritis is the leading cause of septic shock.^9^

In the current study, the foremost 2 antimicrobial agents were beta-lactams (15.2%) and nitrofurantoin (6.4%); and, this was in line with previous researches conducted in Saudi Arabia.^2,10^ High rates of UTIs at the NUH may be related to the beta-lactam antibiotics prescribed there. For asymptomatic bacteriuria and UTIs, beta-lactams are mostly widely prescribed.^11^ Penicillin has been concluded to be safe in pregnancy, and nitrofurantoin is found in the pregnancy category B in the United States.^12^ Moreover, the frequent prescriptions of nitrofurantoin may be due to the frequent genital infections that are pervasive among the participants (26.4%). The current study results corroborate with previous studies carried out in Romania and Ghana, which reported beta-lactam antibiotics to be most commonly prescribed antibiotic during pregnancy.^6,13^

In the current study, the antimicrobial drugs recommended to the participants were of Food and Drug Administration (FDA) category B (26.4%). Our data did not reveal the presence of any class A or class X antibiotics. Similarly, between 2011 and 2015, pregnant women in rural Ghana took antimicrobials predominantly from pregnancy category B (69.6%), with much lower prescription rates for drugs in categories C and D.^6^ The FDA has assigned each medication to one of 5 categories, depending on whether or not data exist on its safety for use during pregnancy, the types of subjects studied, and the findings of those studies.

In our study, antibiotics were suggested in all trimesters, more frequently in the second (19%) and third trimesters (18%). The current study revealed that the antibiotic use during pregnancy was somewhat more in the second trimester (7.2%), followed by the third trimester (3.2%). There have been numerous efforts to encourage the rational use of antibiotics, in part because of the danger of drug resistance, the rising cost of medical care, and the possibility of teratogenicity to the fetus.^14^ Scientific reports, however, have shown that there may be other subtle risks linked to use during pregnancy, and these risks may not be dependent on the safety profile, mechanism of action, or even the class of antibiotic. The use of cesarean sections and the use of intrapartum antibiotics have both been linked to an increased risk of childhood obesity and a weakened immune system.^15^

Almost 3-quarters of the pregnant patients visiting the NUH did not take antibiotics during their pregnancy, and only 13% of the patients took antibiotics for one to 2 weeks, wherein Saudi citizens were more likely (10.4%) compared to other nationalities, as well as pregnant women with a history of miscarriage (7.2%). We found the use of antibiotics to be somewhat higher in Najran, Saudi Arabia, as compared to the use of antibiotics in a German study, wherein approximately 15% of the pregnant women received at least one antibiotic during their pregnancy.^4^

In the current study, 92% of the study participants did not experience any abnormality in lab test reports after using antibiotics, and one of the reasons probably was due to a lower amount of antibiotics usage during pregnancy.

### Study limitations

The single-center study, small sample size, the lack of information of the frequency and type of antibiotics within the class, administered during pregnancy. Finally, it cannot be ruled out that our results could be impacted by lifestyle-related factors other than those that have been reported.

There is currently a dearth of information on the use of antibiotics during pregnancy, and more exploration should be carried out in this area. To guarantee the proper use of antibiotics in this high-risk group, it is advised that healthcare practitioners stay up to date on the latest safety data.

In conclusion, Our findings suggest an association between maternal age and order of pregnancy and the intake of antibiotics during pregnancy. Moreover, an association was observed between maternal BMI and the occurrence of ADRs after antibiotic use. In addition, a history of miscarriage was negatively associated with antibiotic use in pregnancy. Social and lifestyle factors may impact the antibiotic use in pregnancy and childbirth. Antibiotics should be used judiciously; thus, it is important to understand the risk factors that can lead to their misuse. These predictors of antibiotic administration have the potential to serve as general health indicators and to direct preventative strategies aimed at decreasing the inappropriate use of antibiotics.
